# Role of elastography strain ratio and TIRADS score in predicting malignant thyroid nodule

**DOI:** 10.20945/2359-3997000000283

**Published:** 2020-08-24

**Authors:** Hussein Hassan Okasha, Mona Mansor, Nermine Sheriba, Maha Assem, yasmine abd elfattah, Omar A. Ashoush, Maha Rakha, Dalia Abdelfattah, Shereen Sadik El-Sawy, Mai Elshenoufy, Ahmed Amr Mohsen, Heba Kamal Sedrak, Abeer Awad Abdellatif

**Affiliations:** 1 Cairo University Kasr Al-Aini Hospitals Cairo Egypt Kasr Al-Aini Hospitals, Cairo University, Cairo, Egypt; 2 Ain Shams University Cairo Egypt Ain Shams University, Cairo, Egypt; 3 Cairo University National Cancer Institute Biostatistic and Cancer Epidemiology Department Cairo Egypt Biostatistic and Cancer Epidemiology Department, National Cancer Institute, Cairo University, Cairo, Egypt; 4 National Research Centre Cairo Egypt National Research Centre, Cairo, Egypt

**Keywords:** Thyroid nodules, Elastography, TIRADS

## Abstract

**Objective:**

Ultrasonography (US) is the most accurate and cost-effective imaging method in diagnosis of thyroid nodules. A practical thyroid imaging reporting and data system (TIRADS) for thyroid nodules has been proposed to classify nodules of the thyroid gland to solve the problem of nodule selection for fine needle aspiration cytology (FNAC). Real-time elastography and strain ratio (SR) is a method used to assess the stiffness and predict the malignancy of thyroid nodules. The objective of this study was to assess the role of elastography and SR and the TIRADS scoring system in discriminating malignant from benign thyroid nodules.

**Materials and methods:**

From 2015 to 2018 at Cairo University Hospital, a series of 409 patients with thyroid nodules was referred to undergo thyroid ultrasound. Categorization of each nodule according to the TIRADS ranged from 1 to 5. The qualitative elastography score and semiquantitative SR of the nodules were evaluated. Final diagnosis was done by either postthyroidectomy histopathological examination or US-guided FNAC.

**Results:**

Our study included 409 patients with thyroid nodules. Their mean age was 39 ± 10 SD; 36 were males and 373 were females. There were 22 malignant nodules and 387 benign nodules. There were statistical differences between benign and malignant nodules regarding TIRADS classification, SR, anteroposterior/transverse ratio, degree of echogenicity, border, presence of calcification, and absence of halo sign ( *P* < 0.001). The elastic properties of thyroid nodules proved to be a good discriminator between malignant and benign nodules ( *P-* < 0.001) at a cut off value of > 2.32 with 95.2% sensitivity and 86.5% specificity. For every unit increase in SR, the risk of malignancy increased by nearly 2 times. Patients with irregular borders had nearly 17 times increased risk of malignancy than those with regular borders.

**Conclusion:**

Elastography and SR proved to be of high significant value in discriminating benign from malignant nodules, so we recommend adding it to the TIRADS classification.

## INTRODUCTION

Thyroid nodules are widely encountered in the population. The prevalence of thyroid nodules is increasing ( 1 ). They are detected in 2%-6% of the population by palpation, in 19%-35% by ultrasonography (US), and in 8%-65% through autopsy data ( 2 ). US is the most accurate and cost-effective imaging method in diagnosis of thyroid nodules ( 2 ). Ultrasound criteria to differentiate between benign and malignant nodules of the thyroid gland have been the point of research in many scientific papers. Suspicious features of nodules to predict malignancy include hypoechogenicity, microcalcification, taller-than-wide shape, irregular or microlobulated margins, and increased intranodular vascularity ( 3 ).

Fine needle aspiration cytology (FNAC) is mandatory in the preoperative diagnosis of thyroid nodules to distinguish benign from malignant nodules ( 4 ).

Selection of thyroid nodules for FNAC is still confusing. Apractical thyroid imaging reporting and data system (TIRADS) for thyroid nodules has been proposed to classify nodules of the thyroid gland to solve the problem of nodule selection for FNAC ( 5 ).

Real-time elastography is a method used in the evaluation of thyroid nodules by comparing tissue elasticity. Two kinds of elasticity can be assessed by strain elastography. First, colors around and within the nodules are evaluated and visually scored according to the 4-5 scale scoring system. Second, regions of interest are specified as the target region and the adjacent reference region. Later, elastography calculates the strain ratio (SR) automatically. Higher elastography score and SR indicate a high probability of malignancy ( 6 ).

## MATERIALS AND METHODS

### Patients

From 2015 to 2018 at Cairo University Hospital, a series of 409 patients with thyroid nodules (373 females and 36 males) with a mean age of 39 ± 10 SD was referred for thyroid ultrasound. The study was approved by our institution's Research Ethics Committee, and all patients gave their informed written consent before inclusion in the study.

### Thyroid ultrasound

Ultrasound examination of the neck was done using a Hitachi (Avius) machine (Hitachi Medical Corporation). Neck ultrasound examination was done with the patient lying in a supine position, with the neck slightly extended by placing a pillow under the patient's shoulders. The scanning protocol in our study included scanning of the thyroid gland in both transverse and longitudinal planes by brightness mode (B-mode), color-coded Doppler imaging (CCDI) and power Doppler imaging (PDI), and real-time elastography and SR.

### Image analysis

Ultrasound and power Doppler images were analyzed. We did not know the other clinical and final pathological reports; the ultrasound operator was blinded to clinical data and to the pathological results. The thyroid nodules detected in this study were analyzed according to their number (multinodular goiter [MNG] or solitary thyroid nodule), echogenicity (nodules could be “hyperechoic,” “isoechoic,” “hypoechoic,” “marked hypoechoic,” heterogeneous, cystic, or complex cystic), borders (regular or irregular), presence of calcification, and type of calcification (egg shell, microcalcification, or punctate). Other parameters of the nodules were recorded including anteroposterior diameter/transverse ratio (“taller-than-wide,” ≥ 1 mm, and “wider-than-tall,” < 1 mm), presence of breakdown, or a surrounding halo.

**Figure 1:**
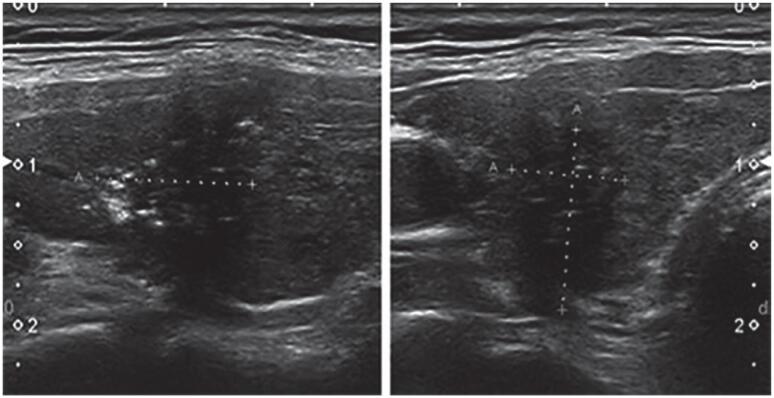
Markedly hypoechoic nodule with irregular outline, AP/T diameter > 1 and microcalcifications (TIRADS 5).

Categorization of each nodule according to the European TIRADS was from 1 to 5: TIRADS1 = normal thyroid gland; TIRADS2 = thyroid gland has a simple cyst, spongiform cyst, or isolated macrocalcification, or is a diffuse hypoechoic enlarged thyroid gland; TIRADS3 = has isoechoic or hyperechoic nodule and has no high suspicious US features; TIRAD4 = has moderately hypoechogenic nodule and has no high suspicious US features; and TIRADS 5 = has at least one of the high suspicious US features and/or adenopathy ( Figure 1 ). Features of high suspicion are irregular shape, irregular margins, microcalcification, and marked hypoechogenicity (and solid) ( 7 ).

**Figure 2:**
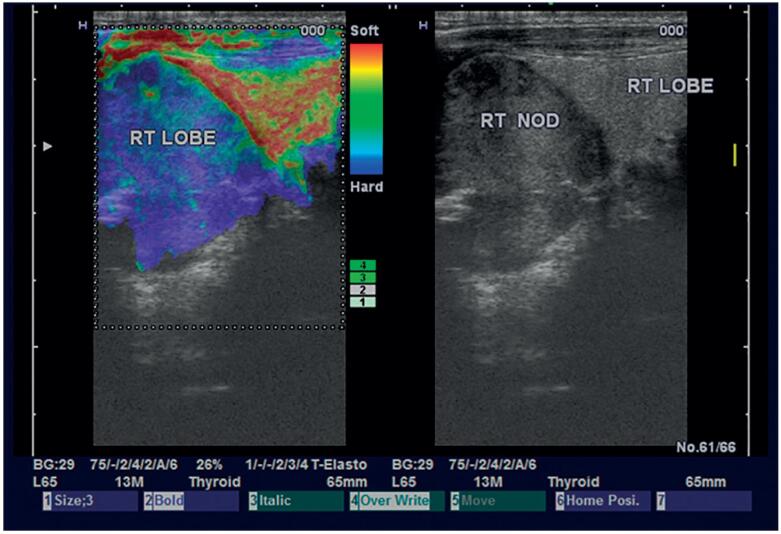
Malignant right lobe nodule with elastography score 4.

Strain elastograms of nodules are qualitatively evaluated with a stepwise scoring system, according to the prevalent color in the nodule. We used the scoring system based on the breast strain USE scale of Itoh and cols. It includes four different patterns. Thyroid nodules with scores 1 and 2 are considered benign, and those with scores 3 and 4 ( Figure 2 ) are classified as suspicious for malignancy ( 7 ).

The semiquantitative score of elastography was represented by the SR method. Two areas were selected: area A, representing the region of interest, and area B, representing the normal area. Area B was then divided by area A. For masses with a homogeneous pattern of elasticity, area A was chosen from any region, but in those with heterogeneous patterns, area A was chosen to cover all heterogeneous areas as much as possible. Both areas were manually selected according to these criteria. Additionally, multiple measures of SR were taken, and the median of these measures was recorded and considered for statistical analysis. Subsequently, the best cutoff value was calculated and was used for the calculation of diagnostic value.

All our data were gathered by a single expert operator, who was blinded to any clinical or cytopathological findings to give the best results and avoid bias.

The final diagnosis was reached by histopathological examination of the thyroid after thyroidectomy in 14% (57/409) and by US-guided FNAC in 86% (352/409).

### Statistical analysis

Data management and analysis were performed using the Statistical Package for Social Sciences (SPSS) version 25. Numerical data were summarized using means and standard deviations or medians and/or ranges, as appropriate. Categorical data were summarized as numbers and percentages. Estimates of the frequency were done using the numbers and percentages. Numerical data were explored for normality using the Kolmogorov-Smirnov test and Shapiro-Wilk test.

To measure associations between categorical variables, a chi-square test or Fisher's exact test was used to compare between two independent percentages.

Odds ratios (ORs) and 95% confidence intervals (95% CIs) were also used (a 95% CI that did not contain 1.0 was considered significant). McNemar's test was used to compare between two dependent percentages. Kappa statistics were used to test for agreement between categorical variables. Comparisons between two groups for normally distributed numeric variables were made using Student's t-test, while for non-normally distributed numeric variables, comparisons were made using the Mann-Whitney U test. Logistic regression was done to give the adjusted OR and magnitude of the effect of different factors. All tests were two tailed, and a p value ≤ 0.05 was considered significant. The receiver operating characteristic curve (ROC curve) was used to determine the best cutoff point, sensitivity, specificity, and area under the curve (AUC).

## RESULTS

Our study included 409 patients with thyroid nodules. Their mean age was 39 years ± 10 SD; 36 were males and 373 were females. Their nodules were classified according to their number into MNG in 323 cases and solitary thyroid nodule in 86 cases. Regarding echogenicity, 47 were isoechoic, 78 were hyperechoic, 257 were hypoechoic, 8 were marked hypoechoic, 14 were heterogeneous, 2 were cystic, and 3 were complex cystic. Calcifications were found in 100 cases: 6 punctate calcifications and 94 microcalcifications. We found 159 cases with breakdown changes inside the nodule and a halo sign in 260 cases. The anteroposterior/transverse ratio was < 1 mm in 398 cases and ≥ 1mm in 11 cases. Real-time elastography revealed that there were 45 cases with score 1, 316 with score 2, 42 with score 3, and 6 with score 4. According to the TIRADS classification, there were 3 TIRADS 2 cases, 100 TIRADS 3 cases, 196 TIRADS 4 cases, and 110 TIRADS 5 cases. The final diagnosis was 22 malignant nodules and 387 benign nodules.

The characteristics of benign (n = 387) and malignant (n = 22) nodules are listed in Table 1 .

**Table 1:** The characteristic of benign and malignant nodules

Factors		Biopsy				*P* value
		Malignant		Benign		
		n = 22	Row %	n = 387	Row %	
Site						
	Isthmic thyroid nodule	0	0.0	9	100.0	1
	Right thyroid nodule	12	5.7	197	94.3	
	Left thyroid nodule	10	5.2	181	94.8	
Elastography						
	1	0	0.0	45	100.0	<0.001
	2	3	0.9	313	99.1	
	3	15	35.7	27	64.3	
	4	4	66.7	2	33.3	
AP/T						
	<1	13	3.3	385	96.7	<0.001
	≥1	9	81.8	2	18.2	
Number of nodule						
	MNG	13	4.0	310	96.0	0.019
	Solitary thyroid nodule	9	10.5	77	89.5	
Nodule character						
	Dominant nodule	11	3.9	271	96.1	0.053
	Small nodule	2	4.9	39	95.1	
	Solitary nodule	9	10.5	77	89.5	
Echogenicity						
	Isoechoic	0	0.0	47	100.0	NA
	Hyperechoic	0	0.0	78	100.0	
	Hypoechoic	14	5.4	243	94.6	
	Marked hypoechoic	8	100.0	0	0.0	
	Heterogenous	0	0.0	14	100.0	
	Cystic	0	0.0	2	100.0	
	Complex cystic	0	0.0	3	100.0	
Border						
	Regular	9	2.3	379	97.7	<0.001
	Irregular	13	61.9	8	38.1	
Calcification						
	No	10	3.2	299	96.8	0.001
	Yes	12	12.0	88	88.0	
Type of calcification 2						
	Microcalcifications	7	7.4	87	92.6	<0.001
	Punctate	5	83.3	1	16.7	
	No	10	3.2	299	96.8	
Breakdown						
	No	17	6.8	233	93.2	0.110
	Yes	5	3.1	154	96.9	
Halo						
	No	19	12.8	130	87.2	<0.001
	Yes	3	1.2	257	98.8	
TIRADS						
	2	0	0.0	3	100.0	NA
	3	0	0.0	100	100.0	
	4	4	2.0	192	98.0	
	5	18	16.4	92	83.6	
	SR					
	Median (range)	5.7 (1.7-9.7)		1.4 (0.1-8.7)		<0.001

P value < 0.05 is considered significant.

No significant differences were determined between the benign and malignant groups in terms of nodule site, number of nodules, and presence of breakdown changes. The anteroposterior/transverse ratio, degree of echogenicity, border, presence of calcification, absence of halo sign, TIRADS classification, and SR were found to be significantly different between benign and malignant nodules as in Table 2 .

**Table 2:** Shows that anteroposterior diameter, the surrounding halo and the calcification can predict malignancy in thyroid nodules

Factors		Malignant		Benign		*P* value	OR	95% CI for OR
		n = 22	(Row %)	n = 387	(Row %)			
AP/T								
	≥1	9	81.8	2	18.2	0.001	133	26-679
	<1	13	3.3	385	96.7			
Calcification								
	Yes	12	12	88	88	0.001	4	1.7-9.8
	No	10	3.2	299	96.8			
Halo								
	No	19	12.8	130	87.2	0.001	12.5	3.6-43
	Yes	3	1.2	257	98.8			

OR = Odds Ratio, 95% CI for OR = 95% confidence interval for the = Odds Ratio. P-value ≤ 0.05 is considered significant.

The elastic properties of thyroid nodules showed to be a good discriminator between malignant and benign nodules (P < .001).

**Figure 3:**
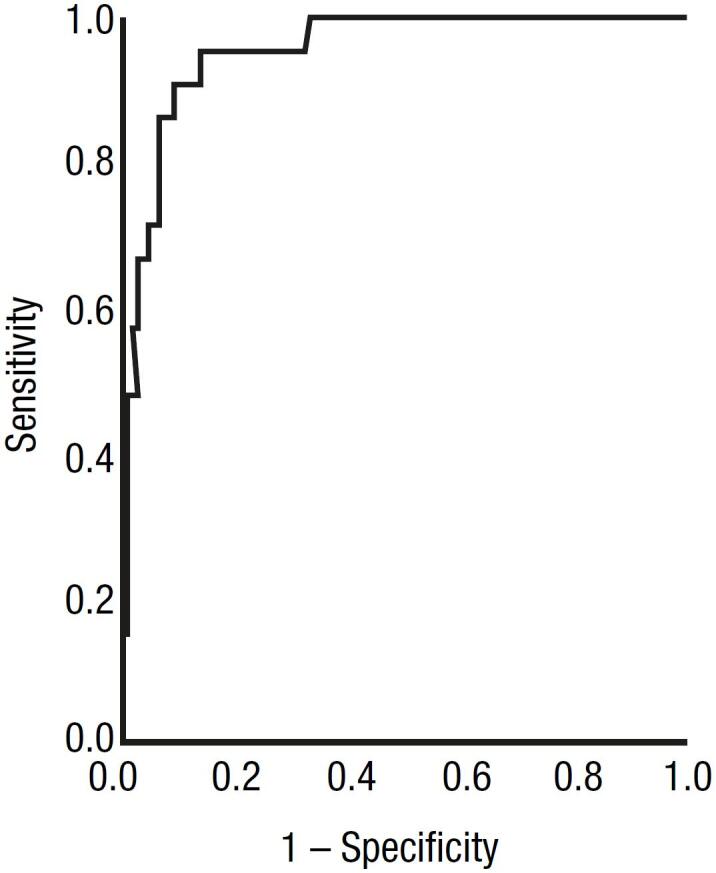
Receiver operating characteristic (ROC) curve analysis to determine cut off point of the strain ratio to discriminate between benign and malignant thyroid nodule.

ROC curve analysis was performed to determine the cutoff point of the SR to discriminate between benign and malignant thyroid nodules ( Figure 3 ). Sensitivity of 95.2%, specificity of 86.5%, 27.8% positive predictive value (PPV), and 99.7% negative predictive value (NPV)were achieved. An AUC of 0.96 and a cutoff value of > 2.32 with *p* value < 0.001 were determined as demonstrated in Table 3 .

**Table 3:** ROC curve to determine cut off point of strain ratio that discriminate between malignant and begin thyroid nodules

Variable	Cut off point	Sensitivity (%)	Specificity (%)	PPV (%)	NPV (%)	AUC	95% CI for AUC	*P* value
SR	> 2.32	95.2	86.5	27.8	99.7	0.96	0.92-0.99	< 0.001

SR: strain ratio; PPV: positive predictive value; NPV: negative predictive value; AUC: area under the curve; CI: confidence interval.

*p* value < 0.05 is considered significant.

### Multivariate analysis (logistic regression for prediction of malignancy in thyroid nodules in Table 4 )

**Table 4:** Multivariate analysis (logistic regression for prediction of malignancy in thyroid nodule)

Factors	B	S.E.	P value	OR	95% CI for OR
SR	0.8	0.2	< 0.001	2.3	1.6-3.4
Border	2.4	0.9	0.009	10.6	1.8-62
Constant	-8.7	1.5	< 0.001	<0.001	

Patients with irregular borders have nearly 17 times increased risk of malignancy than those with regular borders.

For every unit increase in SR, the risk of malignancy increases by nearly 2 times.

## DISCUSSION

US is usually the first chosen modality for diagnosing intra-thyroid lesions. There is no single imaging criterion that can reliably distinguish benign from malignant thyroid lesions ( 8 ).

Currently, ultrasound elastography can evaluate the tissue elasticity of thyroid nodules and thus enhance the diagnostic ability to detect malignant thyroid diseases. Strain elastography determines tissue elasticity, which is related to its composition and cellularity. This is achieved by operator-dependent external compression of the lesion causing tissue deformation. SR is obtained by dividing the mean strain of the surrounding normal tissue by the mean strain within the lesion. Stiff lesions tend to produce high SRs ( 9 ).

The aim of our study was to assess the role of strain elastography and the TIRADS scoring system in discriminating malignant thyroid nodules from benign nodules. It included 409 patients with thyroid nodules, both multinodular lesions and solitary thyroid nodules. No significant differences were determined between the benign and malignant groups in terms of nodule site, number of nodules, and presence of breakdown changes. On the other hand, the anteroposterior/transverse ratio, the degree of echogenicity, the border (with irregular borders showing nearly 17 times increased risk of malignancy), the presence of calcification, the absence of halo sign, and the TIRADS classification were found to be significantly different between benign and malignant nodules.

The results of the current study agree with a series done by Cooper and cols. ( 10 ), who reported that irregular margins, microcalcifications, marked hypoechogenicity, taller-than-wide shape, absent halo sign, and intranodular vascularity are the ultrasonographic features most predictive of malignancy. Furthermore, Mohamed and Abodewan ( 11 ) stated that ill-defined margins, spot microcalcifications, and anteroposterior/transverse diameter more than 1 cm were the most predictive of malignancy.

In our study, the SR was found to be significantly different between benign and malignant nodules, and the elastic properties of thyroid nodules showed to be a good discriminator (P < 0.001). SR with a cutoff value of 2.23 was found to have a sensitivity of 95.2%, specificity of 86.5%, 27.8% PPV, and 99.7% NPV to discriminate between benign and malignant thyroid nodules. Interestingly, we found that for every unit increase in SR, the risk of malignancy increased by nearly 2 times. However, the low specificity might be due to the small number of included malignant nodules (22 nodules), so further studies including a higher number of malignant nodules are recommended.

Our results are in agreement with those of Sachdev and cols., who evaluated 100 thyroid nodules using ultrasound TIRADS classification and performed strain elastography using elasticity score and SR. Strain elastography had high sensitivity and specificity for differentiating malignant from benign thyroid nodules. The majority of benign thyroid nodules had elasticity scores of 1 and 2, while the majority of malignant thyroid nodules had elasticity scores of 3 and 4 ( 9 ). Gay and cols. demonstrated that strain elastography correlated significantly with histological outcomes in indeterminate cytology thyroid nodules ( 12 ).

Bhatia and cols. reported that the difference was most statistically significant using an elasticity score > 2 to predict malignancy, which achieved 75% accuracy (75% sensitivity, 74% specificity) ( 13 ). Rubaltelli and cols., in their study on 40 patients, observed a sensitivity and specificity of 81.8% and 87.5%, respectively, which are slightly higher than those of this study ( 14 ).

Analysis of a database of more than 3000 thyroid nodules that was recorded in a study sponsored by the Society of Radiologists in Ultrasound showed that no more than 2% of TIRADS1 and TIRADS2 nodules, 5% of TIRADS3 nodules, 5% to 20% of TIRADS4 nodules, and at least 20% of TIRADS5 nodules carry malignant risk ( 15 ).

Regarding SR, the results varied. Cantisani and cols. reported that an SR greater than 2 predicted malignancy with a sensitivity of 86% and a specificity of 82%, whereas in another study, Ding and cols. determined a cutoff point of 2.73 with a sensitivity of 89.3% and specificity of 73.2% ( 16 , 17 ).

Dawoud and Dawoud found that gray scale, color Doppler US, and elastography combined were more sensitive and accurate than US features alone in prediction of malignancy of solitary thyroid nodules, with a sensitivity of 94.12%, specificity of 76.74%, and accuracy of 81.67% in 60 patients with solitary thyroid nodules. They also found that the elastography score of malignant nodules (score 3 and 4) was found in 10 (58.8%) of 17 malignant nodules and 9 (20.9%) of 43 benign nodules, with a sensitivity of 58.82%, specificity of 79.07%, and accuracy of 73.33% ( 18 ).

Asteria and cols. ( 19 ) reported that elastosonography had a sensitivity of 94.1%, specificity of 81%, 55.2% PPV, and 98.2%NPVfor thyroid cancer diagnosis, while the accuracy was 83.7%. Rago and cols. ( 20 ) showed sensitivity and specificity as high as 97% and 100%, respectively, using US elastography, while Ferdous and cols. ( 21 ) reported a sensitivity of the elastography score of 84%, while the specificity was 84.7%, the PPV was 70%, the NPV was 92.6%, and the accuracy was 84.5%. Suspicious US findings (TIRADS4 and 5) combined with suspicious elastography score (3 and 4) were found in 16 (94.1%) of 17 malignant nodules, with 94.12% sensitivity, 76.74% specificity, and 81.67% accuracy.

Through logistic regression analysis using 12 different sonographic features of thyroid nodules (margin, border, shape, echogenicity, calcification, posterior acoustic halo, acoustic halo at the periphery of the nodule, capsule of thyroid, vascularity, suspected cervical lymph node metastasis, elastography score, and hypoenhancement pattern), Tiantian and cols. concluded that the conventional US, strain elastography, and contrast enhanced US is an effective and accurate diagnostic tool for differentiating malignant and benign thyroid nodules ( 22 ).

In conflict with our findings, Schenke and Zimny conducted a study including 244 thyroid nodules and analyzed the visual elasticity scores, strain value (SV), and TIRADS classification and found that the sensitivity, specificity, PPV, and NPV of TIRADS were superior to those of sonoelastography. Their data demonstrate that a high TIRADS class alone is predictive for thyroid carcinoma, and the clinical relevance of sonoelastography is negligible ( 23 ). Horvath and cols. reported a sensitivity and specificity for TIRADS classification of 63.64% and 92.31%, respectively ( 24 ). Chandramohan and cols. reported low sensitivity and specificity of sonoelastography score, at 72% and 68%, respectively ( 25 ).

In a study comparing strain elastography to Acoustic Radiation Force Impulse (ARFI) imaging, Georgia and cols. showed that strain elastography had low sensitivity and specificity compared to ARFI (0.86 vs. 0.51, *p* =0.008). The reason could be that the elastography is operator dependent and can be affected by several factors such as the experience of the examiner, characteristics of nodules, and artifacts such as carotid artery pulsations ( 26 ).

In our study, 2 out of 22 malignant nodules had TIRDAS 4 and 5 scores, with longitudinal axes of 14 mm and 10 mm, respectively. They were considered borderline size according to the TIRADS recommendations for FNAC. As they had elasticity scores of 4 and 5 with high SRs (6.78 and 9.68, respectively), they were justified for FNA and not follow-up.

In conclusion, elastography and SR proved to be of high significant value in discriminating benign from malignant nodules, so we recommend adding it to the TIRADS classification. Furthermore, while the TIRADS can be used for selection of high-risk nodules; ultrasound elastography can be employed to differentiate benign nodules from suspicious ones. Subsequently, FNAC can be performed with greater confidence and efficiency. SR will be beneficial in borderline cases, such as in approximately 20 mm TIRADS 3, 15 mm TIRADS 4, or 10 mm TIRADS 5 nodules. FNA will be more favorable than follow-up if such borderline nodules have a high elasticity score or SR above the cutoff level (2.32).
